# Potential Anti-Inflammatory Effects of a New Lyophilized Formulation of the Conditioned Medium Derived from Periodontal Ligament Stem Cells

**DOI:** 10.3390/biomedicines10030683

**Published:** 2022-03-16

**Authors:** Agnese Gugliandolo, Francesca Diomede, Jacopo Pizzicannella, Luigi Chiricosta, Oriana Trubiani, Emanuela Mazzon

**Affiliations:** 1 IRCCS Centro Neurolesi “Bonino-Pulejo”, Via Provinciale Palermo, Contrada Casazza, 98124 Messina, Italy; agnese.gugliandolo@irccsme.it (A.G.); luigi.chiricosta@irccsme.it (L.C.); 2 Department of Innovative Technologies in Medicine and Dentistry, University “G. d’Annunzio” Chieti-Pescara, 66100 Chieti, Italy; francesca.diomede@unich.it (F.D.); oriana.trubiani@unich.it (O.T.); 3 Cardiology Intensive Care Unit, “Ss. Annunziata” Hospital, ASL 02 Lanciano-Vasto-Chieti, 66100 Chieti, Italy; jacopo.pizzicannella@unich.it

**Keywords:** conditioned medium, lyophilization, secretome, mesenchymal stem cell, inflammation, human periodontal ligament stem cells, LPS

## Abstract

The mesenchymal stem cells’ (MSCs) secretome includes the bioactive molecules released in the conditioned medium (CM), such as soluble proteins, free nucleic acids, lipids and extracellular vesicles. The secretome is known to mediate some of the beneficial properties related to MSCs, such as anti-inflammatory, anti-apoptotic and regenerative capacities. In this work, we aim to evaluate the anti-inflammatory potential of a new lyophilized formulation of CM derived from human periodontal ligament stem cells (hPDLSCs). With this aim, we treat hPDLSCs with lipopolysaccharide (LPS) and test the anti-inflammatory potential of lyophilized CM (LYO) through the evaluation of wound closure, transcriptomic and immunofluorescence analysis. LPS treatment increased the expression of *TLR4* and of genes involved in its signaling and in p38 and NF-κB activation, also increasing the expression of cytokines and chemokines. Interestingly, LYO downregulated the expression of genes involved in Toll-like receptor 4 (TLR-4), nuclear factor kappa light chain enhancer of activated B cells (NF-κB) and p38 signaling. As a consequence, the genes encoding for cytokines and chemokines were also downregulated. Immunofluorescence acquisitions confirmed the downregulation of TLR-4 and NF-κB with the LYO treatment. Moreover, the LYO treatment also increased hPDLSCs’ migration. LYO was demonstrated to contain transforming growth factor (TGF)-β3 and vascular endothelial growth factor (VEGF). These results suggest that LYO represents an efficacious formulation with anti-inflammatory potential and highlights lyophilization as a valid method to produce stable formulations of MSCs’ secretome.

## 1. Introduction

Mesenchymal stem cells (MSCs) are multipotent stem cells that represent an attractive approach for cell therapy and regenerative medicine for their beneficial properties, including the differentiation potential and immunomodulatory properties [1]. Dental-derived MSCs were discovered later compared to other MSC types, such as bone marrow MSCs, but they represent a very promising source of MSCs. Indeed, thanks to their easier collection, which did not require an invasive procedure, they attract significant interest [2]. Moreover, they demonstrate excellent differentiation potential, regenerative and immunomodulatory properties and may be expanded with relative genomic stability for a long period of time [3,4,5]. Moreover, dental MSCs derive from the neural crest [6], and for this reason they may possess more potent neurogenic capabilities compared to other MSCs. Therefore, they may have a wide application as treatments for neurological diseases and for neuroregenerative purposes [7,8].

However, it is now well known that the beneficial and regenerative properties of MSCs are also mediated by the soluble factors and vesicles released by MSCs, called the secretome. The secretome includes the bioactive molecules released by MSCs, such as soluble proteins, free nucleic acids, lipids released in the conditioned medium (CM) and extracellular vesicles [9]. The secretome was demonstrated to mediate different beneficial effects, including anti-apoptotic, immunomodulatory and anti-inflammatory activities. Moreover, the secretome was demonstrated to promote tissue repair, exert neuroprotective and neurotrophic actions and antimicrobial effects [9]. 

All these properties were also demonstrated by the dental MSCs-derived secretome. Specifically, the dental MSC-derived secretome was demonstrated to be efficacious for the treatment of neural injuries, for dental tissue and bone regeneration, thanks to its content in anti-inflammatory, anti-apoptotic, osteogenic and neurotrophic mediators [10]. Their CM has also demonstrated anti-inflammatory properties in different models in vivo and in vitro [11,12,13,14].

The use of the secretome presents advantages compared to stem cell therapy, including the lower immunogenicity and easier production, handling and storage [15]. Indeed, CM can be concentrated and frozen, and it is more easily transported than cell-containing materials. Moreover, it does not require liquid nitrogen storage and cell culture facilities [16,17]. 

However, the development of cell-free products, including the CM, as pharmaceuticals, requires the establishment of quality control criteria and the standardization of the manufacturing process, including processes to obtain the desired composition and concentration of CM, its formulation and packaging [16]. Indeed, to be used in clinical practice, the secretome should be transformed into a formulation that is easy to manage and formulated into a standardized pharmaceutical product. 

Lyophilization removes water from a frozen sample by sublimation and desorption. It is a widely used method to produce pharmaceuticals because it increases stability and duration of formulations [18].

In this work, we evaluated the anti-inflammatory efficacy of a novel formulation of CM derived from human periodontal ligament stem cells (hPDLSCs) obtained through lyophilization. With this aim, hPDLSCs were treated with lipopolysaccharide (LPS) and with lyophilized CM (LYO), and the wound healing and transcriptomic profile changes were analyzed. 

## 2. Materials and Methods

### 2.1. Cell Culture Establishment

The present study was performed in accordance with the guidelines of the Helsinki Declaration. The protocol and data collection were approved by the Ethics Committee at the Medical School, “G. d’Annunzio” University, Chieti, Italy (n°266 17 April 2014). The informed consent was obtained from all subjects before sample collection. The Department of Innovative Technologies in Medicine and Dentistry and the Laboratory of Stem Cells and Regenerative Medicine are certified according to the quality standard ISO 9001:2008 (certificate No. 32031/15/S).

Human periodontal ligament biopsies were collected from human premolar teeth of four healthy subjects (donors’ age 18–35 years old, 3 male and Caucasian). The tissue was obtained by scaling the roots using Gracey’s curettes [19]. The samples were washed using the PBS (Lonza, Basel, Switzerland) solution, and placed in a Petri dish with TheraPEAK MSCBM-CD (Lonza, Basel, Switzerland). The medium was changed twice a week, and after two weeks of culture the hPDLSCs spontaneously migrated from the explants. Cells were subcultured until passage 2, when they reached 80% of confluence for the following experiments.

### 2.2. Sample Preparation

The CM from hPDLSCs (15 × 10^3^ cells/cm^2^), cultured in MSCGM-CD, was collected after 72 h of incubation, and centrifuged at 1200 rpm for 5 min. The supernatants were re-centrifuged at 3000 rpm for 3 min, followed by the collection of the secondary supernatants from hPDLSCs [13]. The CM (10 mL) was dried by using a Buchi Lyovapor L-200 (BUCHI Italia s.r.l, Milan, Italy) instrument (condenser capacity 6 kg) operating at a pressure inferior to 0.4 mbar and a capacitor temperature of −55 °C, in order to obtain a lyophilized powder (LYO). 

To quantify the protein, the recovered supernatants (1 mL) were resuspended in ice aceton (3 mL). The obtained solution was maintained at 4 °C for 12 h and then was centrifuged at 16,000 rpm for 12 min at 4 °C (Centrifuge 5804 R, Eppendorf, Milan, Italy). RIPA was used to lysate the suspension and the Breadford assay was used for protein quantization [11]. The total proteins obtained were 125 μg/μL.

In a previous work, we demonstrated that hPDLSCs-CM contained 378.77 pg/mL of interleukin (IL)-10, 748.58 pg/mL of stromal cell-derived factor (SDF)-1α and 451.45 pg/mL of transforming growth factor (TGF)-β [12]. Given that 10 mL of CM were lyophilized and the obtained powder was resuspended in 1 mL, we can suppose that in LYO, the concentrations of these cytokines are: IL-10, 3787.7 pg/mL, SDF-1α 7485.8 pg/mL and TGF-β 4514.5 pg/mL.

### 2.3. Evaluation of Cell Metabolic Activity to Determine the LYO Concentration

Human PDLSCs were seeded at a cell density of 2000 cells/well into a 96-well tissue culture plate. The cell metabolic activity of hPDLSCs was evaluated at the established time points: 24, 48 and 72 h of treatment, with the following concentration of LYO resuspended in MSCBM: 0.5 μg/μL, 1 μg/μL, 2 μg/μL and 4 μg/μL as well as for the sample groups reported in the following section, namely, the “study design”.

At each endpoint, 20 μL of MTS (CellTiter 96^®^ Aqueous One Solution Cell Proliferation Assay, Promega, Madison, WI, USA) solution was added to each well. Samples were maintained for 3 h at 37 °C. The formazan production was identified by absorbance measurements at a 490-nM wavelength by means of the Synergy™ HT Multi-detection microplate reader (Biotech, Winooski, VT, USA). The assay was performed in three independent experiments. 

### 2.4. Study Design

The following experiments were performed using the isolated hPDLSCs at passage 2. Here, we reported the experimental groups:

- Untreated hPDLSCs, used as negative control (CTR);
- hPDLSCs treated with lyophilized conditioned medium resuspended in MSCBM-CD (Lonza) at a concentration of 2 μg/μL (LYO);
- hPDLSCs treated for 24 h with ultrapure LPS from *Porphyromonas gingivalis* (tlrl-ppglps, InvivoGen, San Diego, CA, USA), 5 μg ml^−1^ (LPS);
- hPDLSCs treated for 24 h with LPS and LYO (LPS + LYO).
- All samples were observed at inverted light microscopy DMIL (Leica Microsystem, Milan, Italy) to evaluate the morphological cell features.

### 2.5. Wound Healing Assay

A wound-healing analysis was executed to assess the percentage of wound closure after the treatments [20]. 

Human PDLSCs were cultured in a concentration of 3 × 10^5^ cells/dish. Twenty four hours before the treatment, cells were maintained in DMEM (Lonza) for cellular starvation. A sterile pipette with 10 µL tip was used to wound the cell monolayer. At defined time points after wounding (2 h, 6 h, 24 h and 48 h), an inverted light microscope (DMIL, Leica Microsystem, Milan, Italy) was used to analyze the rate of wound closure, calculating the migrated distance/total wound distance using the LEICA LAS/EZ software (version 3.4, Leica Microsystem, Milan, Italy).

### 2.6. Transcriptomic Inspection

At the end of the treatment, RNA was extracted from CTR, LPS and LPS + LYO groups using the Maxwell^®^ RSC simplyRNA Cells Kit (Promega, Milan, Italy) according to the manufacturer’s instructions. The library preparation was performed following the TruSeq RNA Exome protocol (Illumina, San Diego, CA, USA) following the instructions, as previously described [21].

The quality check of the raw data sequenced using the MiSeq instrument of Illumina was confirmed using the tool, fastqc version 0.11.4 (Babraham Institute, Cambridge, UK). Then, the adapters and all the bases with poor quality score were filtered out using Trimmomatic version 0.38 (Usadel Lab, Aachen, Germany) [22], and the cleaned reads were aligned with the Spliced Transcripts Alignment to a Reference (STAR) RNA-seq aligner 2.7.3a (New York, NY, USA) [23] and to the GRCh38 human reference genome. The aligned reads were used to compute the counting of the transcripts through the python package htseq-count version 0.6.1p1 (European Molecular Biology Laboratory (EMBL), Heidelberg, Germany) [24]. The differentially expressed genes (DEGs) were then computed in the R version 3.6.3 (R Core Team) through the library, DESeq2 [25]. We removed the false positive DEGs using the post hoc Benjamini–Hochberg procedure, setting the q-value to 0.05. Thus, we avoided using a fold change cut-off. In order to inspect the biological role of LPS and LYO, we used the “Toll-like receptor signaling pathway” (hsa04620) and the “NF-kappa B signalling pathway” (hsa04064) collected on the KEGG website (https://www.genome.jp/kegg/ (accessed on 10 January 2022)) [26]. In addition, we used the HUGO Gene Nomenclature Committee (HGNC) database [27] to inspect the inflammatory markers “Chemokine ligands” (ID group 483) and “Interleukins” (ID group 601).

### 2.7. Immunofluorescence Analysis and Confocal Laser Scanning Microscopy (CLSM) Analysis

All samples were processed for the visualization to CLSM. A 4% solution of paraformaldehyde in 0.1 M PBS (Lonza, Basel, Switzerland) was used to fix the cells. Then, the permeabilization process was performed by means of the use of 0.5% Triton X-100 in PBS (Lonza) for 10 min, followed by blocking with 5% skim milk in PBS (Lonza) for 30 min. Subsequently, the cells were incubated for 2 h at room temperature with the following primary antibodies: anti-TLR4 (1:500, SantaCruz Biotechnology, Dallas, TX, USA) and anti-NF-κB (1:200; SantaCruz Biotechnology). Red fluorescent Alexa Fluor 568 (1:200; Molecular Probes, Thermo Fisher Scientific, Waltham, MA, USA) dye, labeled as the secondary antibody, was used with an incubation for 1 h at 37 °C. The filamentous actin was stained with Alexa Fluor 488 phalloidin (1:400; Molecular Probes, Eugene, Oregon) green-fluorescent, and the nuclei were counterstained with TOPRO (1:200; Molecular Probes) conjugated blue fluorescence. The samples were mounted on glass coverslips using Pro-Long Gold Antifade (Molecular Probes). All pictures were captured at CLSM (LSM800, Zeiss, Jena, Germany) with a resolution of 1024 × 1024 pixels at 12 bit (4096 grey values) and extracted with ZEN 3.0 SR software (Zeiss, Jena, Germany).

### 2.8. Western Blot Analyses

Proteins were extracted from hPDLSCs, CM and LYO samples. The proteins were extracted from hPDLSCs, CM and LYO samples of the same set of experiments. Specifically, hPDLSCs were cultured, as reported in the Section 2.1; the cells were resuspended in a RIPA cold hypotonic lysis buffer (1× PBS, 1% Igepal, 0.5% sodium deoxycholate, 0.1% sodium dodecyl sulphate (SDS) and 10 µg/mL phenylmethylsulfonyl fluoride (PMSF), 10 µg/mL leupeptin and 10 µg/mL soybean trypsin inhibitor as inhibitors). The level of recovered protein was measured spectrometrically, according to the instructions of the manufacturer using the Bio-Rad (Hercules, CA, USA) Protein Assay (detergent compatible). To recover proteins from CM and LYO, derived from hPDLSCs as described in the Section 2.2, cold acetone was added to the protein solution (4:1 volume) and then was maintained at 20 °C overnight. The solution was centrifuged for 15 min at 15,000× *g*. The supernatant was discharged and the pellet was resuspended in the RIPA buffer. Subsequently, the proteins were separated using sodium dodecyl-sulfate polyacrylamide gel electrophoresis (SDSPAGE) followed by Western blot analysis (Bio-Rad V3 Western Workflow™, Milan, Italy). The membranes were saturated for 120 min at room temperature in a blocking buffer (1× TBS, 5% milk, 0.1% Tween-20), followed by overnight incubation at 4 °C with the following primary antibodies: mouse anti-vascular endothelial growth factor (VEGF) (1:350; SantaCruz Biotechnologies, Santa Cruz, CA, USA), mouse anti-TGFβ3 (1:350; SantaCruz Biotechnologies) and anti-β actin (1:750; SantaCruz Biotechnologies), as the housekeeping protein. Subsequently, the membranes were incubated for 60 min at room temperature with peroxidase-conjugated anti-mouse secondary antibody (1:5000; Bethyl Laboratories, Montgomery, AL, USA). An enhanced chemiluminescence with the Alliance 2.7 system (Uvitec Ltd., Cambridge, UK) was used to identify and quantify the obtained bands.

### 2.9. Statistical Analysis

The statistical analysis was executed by means of the GraphPad Prism software (version 5.01, GraphPad Software, San Diego, CA, USA). To establish the statistically significative differences, the one-way ANOVA, followed by Tukey’s post hoc test, were used. A *p*-value less than or equal to 0.05 was considered statistically significant. Data were presented as the mean ± S.E.M.

## 3. Results

### 3.1. Cell Metabolic Activity to Determine the LYO Concentration

**Figure 1:**
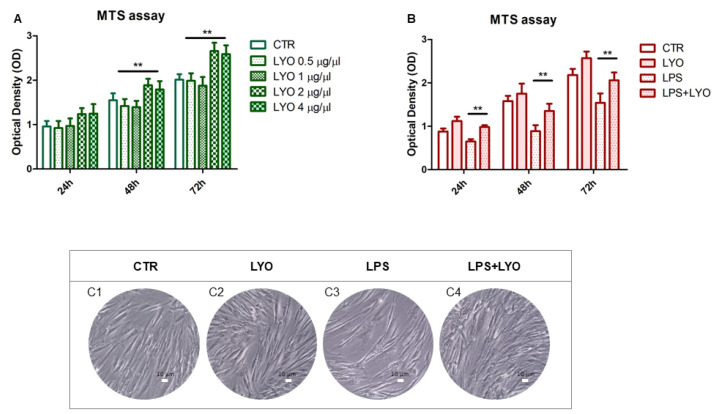
Graph bar of cell metabolic activity at defined time points 24, 48 and 72 h of culture in (**A**) human periodontal ligament stem cells (hPDLSCs) treated with different concentrations of LYO and in (**B**) hPDLSCs treated with lipopolysaccharide (LPS)/LYO alone or in co-treatment. No cytotoxicity was found for all the LYO concentrations tested. Moreover, in order to evaluate whether LYO was able to rescue LPS-induced loss of metabolic activity, we incubated cells with LPS + LYO and performed MTS assay. Results demonstrated that LYO was able to revert the loss of cell metabolic activity induced by LPS. The results are reported as the mean (±S.E.M.) of three independent experiments. ** *p* < 0.01. Inverted light microscopy pictures of (**C1**) hPDLSCs under basal medium (CTR), (**C2**) hPDLSCs treated with selected concentration of LYO (LYO), (**C3**) hPDLSCs treated with LPS and (**C4**) hPDLSCs treated with LPS/LYO. Scale bar = 10 µm.

To choose the best concentration of LYO, the MTS was performed at: 0.5 μg/μL, 1 μg/μL, 2 μg/μL and 4 μg/μL. Human PDLSCs demonstrated a logarithmic rate for metabolic activity after 24, 48 and 72 h of culture. Cells treated with LYO at a concentration of 0.5 μg/μL and 1 μg/μL demonstrated no statistically significant differences in all considered time points when compared to the CTR group. Human PDLSCs treated with 2 μg/μL and 4 μg/μL demonstrated a high performance in terms of metabolic activity in all considered end points, especially at 48 and 72 h of culture (Figure 1A). The following experiments were performed with LYO at a concentration of 2 μg/μL.

### 3.2. LYO Treatment Protected against the LPS Stimulus in hPDLSCs Cell Metabolic Activity

The effects of LYO alone or in combination with LPS were evaluated by means of MTS assay on hPDLSCs’ culture. As shown in Figure 1B, the bar graph reported a logarithmic trend of the cell metabolic activity of the CTR group; the same trend is demonstrated by cells treated with LYO. In all considered time points, the LPS stimulus demonstrated a decrease in the metabolic activity when compared to the other culture conditions. The cell metabolic activity was similar to the CTR group in cells co-treated with LPS and LYO (Figure 1B). The CTR group observed under light microscopy showed a classical fibroblastoid cellular shape with numerous elongated processes Figure 1(C1). Human PDLSCs treated with LYO showed no morphological differences when compared to the CTR group Figure 1(C2). The LPS treatment showed some morphological changes and the presence of translucent cells, while the treatment with LPS/LYO showed a restored morphology in hPDLSCs similar to the CTR sample Figure 1(C3,C4).

### 3.3. LYO Treatment Produced Migration of hPDLSCs Stimulated by LPS

**Figure 2:**
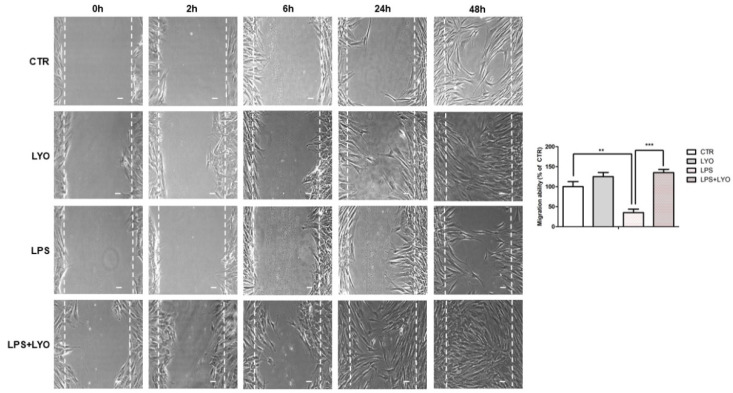
Confluent cell cultures for all conditions were wounded after treatment, and migration distances were assessed at 2 h, 6 h, 24 h and 48 h. The results shown in the graph are data from all experimental groups at 48 h, reported as the percentage of the migration capability towards the control cells and measured using the LEICA LAS/EZ software (3.4) (CTR, 100%; means SD, *n* = 3). The images are representative of three independent experiments. ** CTR vs. LPS: *p* < 0.01; *** LPS vs. LPS + LYO: *p* < 0.001. Scale bar = 10 µm.

A wound-healing analysis was executed to investigate the ability to migrate of hPDLSCs cultured in different conditions. The quantitative analysis was carried out by measuring the migrated distance/total wound space, and expressed as the percentage of untreated cells (CTR). After 24 h of treatment with LYO and LPS + LYO, an augmentation of cell migration occurred, and after 48 h of treatment, the wound completely closed. In LPS-treated cells, the migration was slow when compared to the other experimental groups (Figure 2). 

### 3.4. Transcriptomic Analysis

The analysis of the comparisons of CTR against LPS and LPS against LPS + LYO revealed 9261 and 4476 DEG, respectively. In detail, we observed 4875 upregulated and 4386 downregulated DEGs in the CTR against the LPS comparison, whereas 2211 upregulated and 2265 downregulated DEGs in LPS against the LPS + LYO groups. 

For each of the two comparisons, we evaluated the DEGs in the “Toll-like receptor signaling pathway” and the “NF-kappa B signaling pathway” using KEGG (Table 1) and focusing on Toll-like receptor 4 (TLR-4) signaling. We found 21 genes upregulated and two genes downregulated when CTR was compared to the LPS group. Instead, 14 downregulated and three upregulated genes were observed in the comparison of LPS against LPS + LYO.

Moreover, we searched for inflammatory markers collected in the HGNC database identified as “Chemokine ligands” and “Interleukins” (Table 2). All the genes were upregulated in the comparison of CTR against LPS. Instead, all the genes were downregulated when the LPS group was compared to the LPS + LYO group.

### 3.5. Reduction of TLR-4 and NF-κB after LYO Treatment

**Figure 3:**
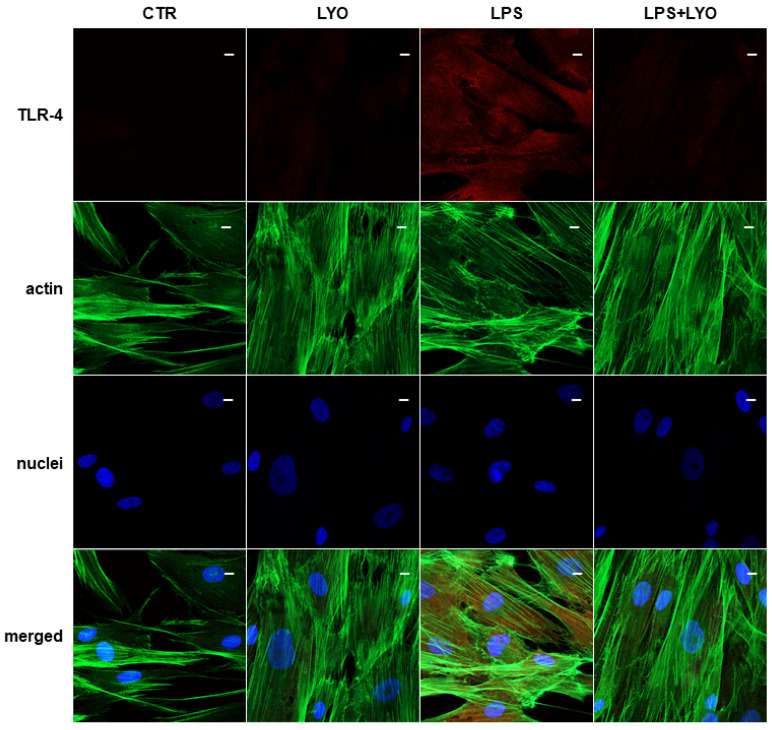
(CTR) Untreated cells show a negative expression for Toll-like receptor 4 (TLR-4). (LYO) Cells treated with LYO show a negative expression for TLR-4. (LPS) Cells stimulated with LPS show a positive expression for TLR-4. (LPS + LYO) Cells treated with LPS and LYO show a slight fluorescence signal for TLR-4. Green fluorescence derived from Alexa-phalloidin 488 staining for cytoskeleton actin; red fluorescence derived from Alexa Fluor 568-IGg conjugated secondary antibody to reveal primary antibody against TLR-4; blue fluorescence derived from TO-PRO staining of nuclei. Scale bar = 20 µm.

Images acquired using a confocal microscope showed the expression of TLR-4 in hPDLSCs treated with LPS derived from *Prophyromonas gingivalis* (Figure 3). 

**Figure 4:**
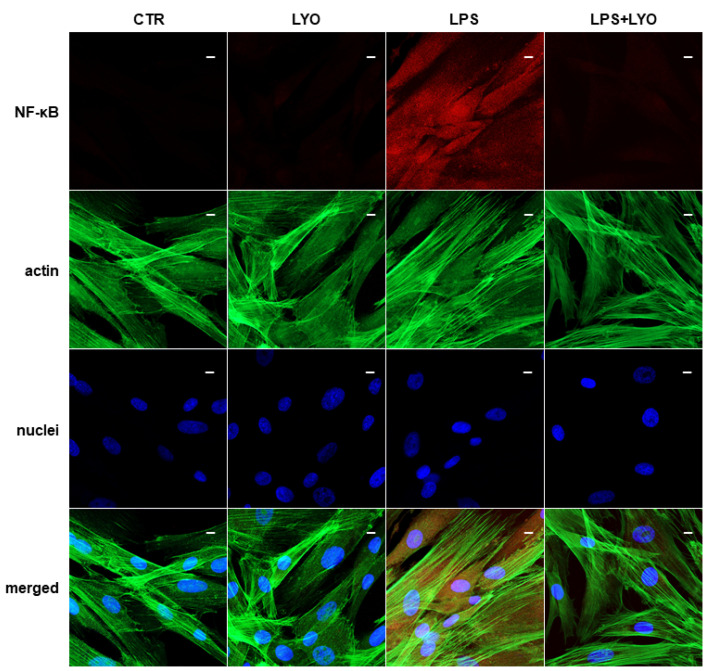
(CTR) Untreated cells show a negative expression for nuclear factor kappa light chain enhancer of activated B cells (NF-κB). (LYO) Cells treated with LYO show a negative expression for NF-κB. (LPS) Cells stimulated with LPS show a positive expression for NF-κB. (LPS + LYO) Cells treated with LPS and LYO show a slight fluorescence signal for NF-κB. Green fluorescence derived from Alexa-phalloidin 488 staining for cytoskeleton actin; red fluorescence derived from Alexa Fluor 568-IGg conjugated secondary antibody to reveal primary antibody against NF-κB; blue fluorescence derived from TO-PRO staining of nuclei. Scale bar = 20 µm.

The inflammation pathway is also sustained by the nuclear factor kappa light chain enhancer of activated B cells (NF-κB) activation, as demonstrated in the LPS-treated cells in Figure 4.

The treatment with LYO showed a beneficial effect in LPS-stimulated cells. Cells co-treated with LPS + LYO showed a reverting expression for TLR-4 and NF-κB, in a similar level as the CTR samples (Figure 3 and Figure 4, LPS + LYO panel). 

### 3.6. Evaluation of Genes Involved in MSC Migration

We also evaluated genes already associated with MSCs’ migration in literature, with some of them belonging to the KEGG “ECM-receptor interaction” pathway, such as the extracellular matrix components and integrins, or growth factors (Table 3), which may support the differences in cell migration between the groups, as suggested by the wound-healing analysis.

### 3.7. Analysis of Growth Factors in LYO

**Figure 5:**
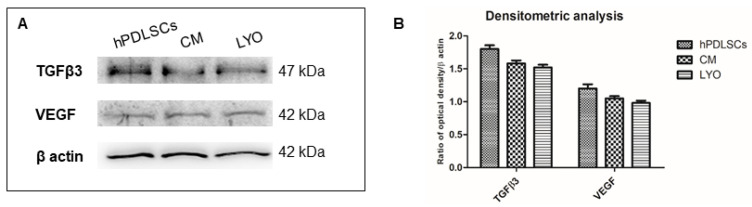
(**A**) Specific bands of TGF-β3 and VEGF obtained from human periodontal ligament stem cells (hPDLSCs), conditioned medium (CM) and lyophilized CM (LYO). The proteins were extracted from hPDLSCs, CM and LYO samples of the same set of experiments. (**B**) Bar graph of densitometric analysis.

We explored the growth factors present in the hPDLSCs, CM and LYO. Western blotting was used to detect the protein expression levels of VEGF and TGF-β3 (Figure 5). The bar graph revealed similar expression levels of VEGF and TGF-β3 in all samples.

## 4. Discussion

MSCs’ secretome was demonstrated to exert beneficial effects and presents advantages compared to cell-therapy. In particular, its storage and handling may be easier compared to cell therapy. Freeze-drying, or lyophilization, is a widely used process to improve the long-lasting stability of formulations. Different biological products are freeze-dried, such as small molecules, heat-sensitive pharmaceuticals and biologically active molecules, including enzymes, hormones, antibiotics and vitamins. Sometimes additives, such as protective agents or stabilizers, can be added, if during the procedure, the product undergoes different stresses that can influence the end quality of the formulation [28,29]. 

In this work, we evaluated the anti-inflammatory potential of a new formulation obtained by the lyophilization of CM derived from hPDLSCs. We evaluated the anti-inflammatory potential in hPDLSCs treated with LPS using multiparametric analyses. 

At first, in order to define the best concentration of LYO, the MTS was performed using four different LYO concentrations (0.5 μg/μL, 1 μg/μL, 2 μg/μL and 4 μg/μL). While cells treated with LYO at the concentrations of 0.5 μg/μL and 1 μg/μL were similar to the controls, hPDLSCs treated with 2 μg/μL and 4 μg/μL demonstrated a higher metabolic activity at all considered time points. Moreover, the highest LYO concentration demonstrated similar results to the concentration of 2 μg/μL. For this reason, this latter dose was chosen for the other experiments. 

A previous work evaluated the effects on the proliferation of hPDLSCs exposed for 48 h to different lyophilized CM concentrations. A higher proliferation was found when cells were treated with 6.25 mg/mL CM, while higher concentrations inhibited the proliferation of hPDLSCs. When cells were treated with lower concentrations (range of 3 mg/mL–1.5 μg/mL), the proliferation rate was not significantly different from the control cells [30].

LPS stimulation leads to a higher expression of several cytokines related to the pro-inflammatory cascade, including interleukin (IL)-1β, tumor necrosis factor-alpha (TNF-α) and IL-6, which represents the trigger point for periodontal tissue destruction [31,32]. LPS stimulation also promotes a reduction in cell proliferation, cell migration, and cell adhesion of hPDLSC culture [33]. However, our data demonstrated that the LYO treatment could restore a cell metabolic activity similar to the untreated cells.

Wound healing is a physiological process, which leads to tissue regeneration. The wound healing in vitro test is adapted to evaluate cell migration and the capability to lead a wound closure. The LPS treatment attenuates the cell proliferation, cell migration, and cell adhesion in hPDLSCs [33]. As reported, the capacity of MSCs to provide regeneration is a secretome-based process, which has become more common as a strategy to discover novel therapeutic targets [9]. In our in vitro study, the LYO treatment demonstrated a beneficial effect in the wound-healing assay when cells were stimulated with LPS. These data indicated that LYO exposure was able to increase hPDLCSs’ migration. This effect may be due to the presence in the CM of SDF-1α and TGF-β, as previously demonstrated [12], which are known to act as chemoattractants for MSCs [34,35]. Integrins, extracellular matrix components and growth factors can regulate MSCs’ migration and homing [36,37]. We found the upregulation of *ITGA4* and *ITGB1*, encoding for the α4 and β1 integrin. Their increased expression is also associated with the increased migration of MSCs [38,39]. Moreover, the integrins α4 and β1 have an important role in the transmigration and homing process, also through the interaction with fibronectin [40,41]. It was reported that MSC migration is increased by fibronectin, involving PDGFR-β [42]. Interestingly, we found the *FN1* gene, encoding for fibronectin, upregulated in the group LPS + LYO, and *PDGFRB* also showed an increased expression, along with *PDGFA* and *PDGFD*, which may increase the migration of MSCs [43]. We also found a downregulation of the integrin α2 subunit encoded by *ITGA2*. A decrease in its levels was also reported in hypoxic preconditioned MSCs, which demonstrated an enhanced migration [44]. We can speculate that MSCs increased the fibronectin, which increased the migration through the integrin signaling. We also found a differential expression of genes encoding for THBS (*COMP*, *THBS1*, *THBS2*, *THBS3* and *THBS4*) that may also be involved in MSC migration [45]. 

Our group has already demonstrated that the treatment of hPDLSCs with LPS increased TLR-4 and NF-κB. As a consequence, an increase in the cell culture medium of the pro-inflammatory cytokines IL-1α, IL-8, IL-15, Eotaxin, Eotaxin 2, M-CSF, TGF-β1, TNF-α and TNF-β was also found. On the contrary, the anti-inflammatory cytokine IL-10 was reduced after LPS treatment [46]. In addition, our NGS analysis revealed the up-regulation of TLR-4 signaling in hPDLSCs treated with the LPS as compared to the control, in association with the increased expression of cytokines. Specifically, we found the upregulation of *TLR4* and *LY96* encoding for MD-2, which plays a role in responsiveness to LPS through the binding to LPS and to the extracellular domain of TLR-4 [47]. The transcriptomic profile also evidenced the upregulation of *IRAK1*, which is essential for the NF-κB activation in TLR signaling [48], *TRAF6* and TAK1, encoded by *MAP3K7*. *TAB1* and *TAB2* were downregulated, but they are not necessary for TLR signaling [49,50]. As a consequence of the TLR4 activation, NF-κB and p38 signaling appeared to be activated. In particular, the IKK subunits, *CHUK* and *IKBKB,* were upregulated. We also found the upregulation of the inhibitor *NFKBIA*, but this depended on a negative feedback regulation. The genes, *MAP2K3*, encoding for MKK3, and *MAPK11*, *MAPK12* and *MAPK14*, encoding for p38, were upregulated. The activation of these pathways led to the upregulation of the genes encoding for the cytokines IL-6 (*IL6*), IL-12 (*IL12A*), IL-8 (*CXCL8*) and RANTES (*CCL5*). We also looked at the expression of TRIF encoded by *TICAM1*, given that it is essential for the TLR-4-mediated activation of the MyD88-independent pathway [48], and it was upregulated. It is known that LPS can trigger the MyD88-independent pathway, activating IRF3 and leading to the production of IP-10 [51]. Indeed, we also found the upregulation of *TRAF3*, *IKBKE*, *IRF3* and *CXCL10*, which encodes for IP-10.

Interestingly, the LYO treatment reduced the *TLR4* expression. The reduction of TLR-4 signaling, as a protective and anti-inflammatory mechanism exerted by MSCs’ CM, was reported in different models in vitro and in vivo [11,12,52]. In addition, the genes *LY96* and *TIRAP*, which participate in TLR4 signaling [48], were found to be downregulated in our analysis. Furthermore, after the LYO treatment, *IRAK1* was upregulated, even if it was with a lower fold change. However, *TOLLIP* was also upregulated, which binds to IRAK1 and inhibits TLR-mediated responses [53]. As a consequence of the downregulation of TLR-4 signaling, NF-κB and p38 signaling were also downregulated. Specifically, the subunit *RELA* of NF-κB was found downregulated, while the inhibitor IκBα, encoded by the gene *NFKBIA,* was upregulated. The genes *MAP2K3* and *MAPK13*, encoding for MKK3 and p38, respectively, were both downregulated. In accordance with the downregulation of NF-κB and p38, the genes encoding for the cytokines IL-6, IL-12, IL-8 and RANTES, but also COX2 (*PTGS2*), were all downregulated. *TICAM1* was downregulated, together with *TRAF3* and *CXCL10*, also indicating the downregulation of the MyD88-independent pathway. 

We also looked at cytokines’ and chemokines’ expression. Treatment with LPS increased cytokines’ and chemokines’ expression (*CCL11*, *CCL2*, *CCL26*, *CCL28*, *CCL5*, *CXCL1*, *CXCL10*, *CXCL16*, *CXCL8*, *IL12A*, *IL24*, *IL32* and *IL6*). We observed the downregulation of different chemokines and cytokines after the LYO treatment. Genes encoding for the chemokines, eotaxin (*CCL11*), eotaxin 3 (*CCL26*) and IP-10 (*CXCL10*) and for cytokines IL-24 and IL-32, were downregulated. An immunofluorescence analysis validated the expression of TLR-4 and NF-κB, and, indeed, the inflammatory molecules were down-regulated in hPDLSCs that were co-treated with LPS + LYO.

These cytokines and chemokines are known to mediate macrophage responses. IL-32 is known to induce the production of IL-8 and TNF-α in monocytes and macrophages [54], also promoting the differentiation of monocytes into macrophages [55]. IL-6 is widely produced by macrophages and has a role in their polarization [56]. IL-24 also has a role in the macrophages’ polarization and can promote their renewal. It was also demonstrated to induce monocyte migration in vitro, indicating its ability to modulate the immune response, as a chemoattractant [57]. IL-12 is abundantly produced by macrophages, activates natural killer cells and induces the differentiation of T lymphocytes [58]. CCL2 is one of the main chemokines that mediates the migration and infiltration of monocytes and macrophages [59]. CCL5 has a role in the macrophages’ recruitment and infiltration [60,61]. CXCL8 and CXCL10 were reported to induce the M1 macrophage chemotaxis [62].

We have also already demonstrated in previous studies that hPDLSCs-CM contain anti-inflammatory molecules, such as IL-10 and TGF-β [11,12,13]. We performed a Western blot analysis to evaluate the content of VEGF and TGF-β3 in hPDLSCs, CM and LYO. We found their presence in all three samples. Moreover, the content of these growth factors in CM and LYO was similar, suggesting that the lyophilization procedure did not alter the growth factor content of the CM. These growth factors mediate different beneficial effects of the CM. Both VEGF and TGF-β were reported to increase the MSCs’ migration [34,35,43,63,64,65,66,67] and proliferation [65,67,68]. Sakaguchi et al. created a cocktail mimicking CM, also containing VEGF and TGF-β, and demonstrated that this mix was able to increase MSC migration [69]. The presence of VEGF and TGF-β3 may explain the increased migration observed after the treatment with LYO. Moreover, these growth factors mediate the different protective and beneficial actions of MSCs and their secretome, and their presence can explain the overall potential of LYO. Indeed, the presence of TGF-β3 can support the anti-inflammatory potential of LYO. It was reported that dental follicle stem cells CM-containing TGF-β3 was able to exert an anti-inflammatory response and to elicit macrophage M2 polarization [70]. We have already reported that the pre-treatment with hPDLSC-CM, containing TGF-β, inhibits the LPS-induced TLR4 and NF-κB activation in NSC34 motoneuron cells that are exposed to LPS [11]. VEGF, produced by MSCs, was demonstrated to mediate, at least in part, their beneficial and neuroprotective effects [71], but VEGF is also a well-known angiogenic factor.

These results indicated that these factors may be responsible for the increased migration and the anti-inflammatory effects of hPDLSCs-CM; these soluble factors also maintained their role and content after lyophilization. A previous study demonstrated that lyophilized CM derived from corneal MSCs was as effective as fresh CM in promoting wound healing and modulating inflammation [72]. 

However, lyophilization may be helpful to control the concentration after reconstitution, in order to use lyophilized CM for many experiments for many months with consistent results, and also using the same batch of lyophilized CM [73]. Then, on the basis that LYO demonstrated the same content of growth factors as CM, LYO demonstrated a better application in the therapeutic contest when compared to CM. In particular, the advantage of LYO is regarding the easier storage in stable conditions.

In particular, it is necessary to consider the limitation caused by the variability of the content and concentration of the growth factors of the CM, which are linked to different donors of MSC. In particular, CM is known to differ in relation to the age of the dental stem cell donors. It is known that stem cells derived from permanent teeth produce CM with different growth factors compared to those obtained from deciduous teeth [74,75]. For this reason, it is important to carry out a characterization of the CM and the lyophilized formulation.

## 5. Conclusions

Our results support the efficacy of lyophilized CM derived from hPDLSCs as a new formulation with anti-inflammatory potential and with the ability to increase MSC migration. In addition, the lyophilization process did not alter CM content in growth factors and, consequently, the beneficial effects. Then, lyophilization may be considered as an appropriate method to produce a more stable and long-standing formulation of MSCs’ secretome. However, CM represents a cocktail of different molecules then a deep investigation into its nature is needed to discover the overall potential of CM.

## Figures and Tables

**Table 1:** Differentially expressed genes in the KEGG maps “Toll-like receptor signaling pathway” and “NF-kappa B signaling pathway” between control cells and human periodontal ligament stem cells (hPDLSCs) treated with lipopolysaccharide (LPS) and between hPDLSCs treated with LPS, compared to hPDLSCs treated with LPS and LYO.

Gene	CTR vs. LPS Fold Change	CTR vs. LPS *q*-Value	LPS vs. LPS + LYO Fold Change	LPS vs. LPS + LYO *q*-Value
*CCL5*	4.59	1.98 × 10^−5^	−1.51	1.97 × 10^−5^
*CHUK*	0.83	1.10 × 10^−21^	-	>0.05
*CXCL8*	7.21	1.66 × 10^−6^	−2.39	8.73 × 10^−9^
*CXCL10*	5.60	2.55 × 10^−4^	−1.78	6.58 × 10^−3^
*IL12A*	7.97	1.04 × 10^−7^	−1.98	9.80 × 10^−13^
*IL6*	4.95	3.69 × 10^−11^	−0.61	6.27 × 10^−4^
*IKBKB*	0.55	5.90 × 10^−8^	-	>0.05
*IKBKE*	1.98	1.86 × 10^−8^	-	>0.05
*IRAK1*	1.04	2.95 × 10^−53^	0.14	7.25 × 10^−3^
*IRF3*	0.60	6.01 × 10^−3^	-	>0.05
*LY96*	1.82	1.02 × 10^−15^	−0.84	5.32 × 10^−8^
*MAP2K3*	1.02	9.99 × 10^−33^	−0.32	1.66 × 10^−6^
*MAP3K7*	0.20	6.49 × 10^-3^	-	>0.05
*MAPK11*	1.50	2.69 × 10^-2^	-	>0.05
*MAPK12*	0.88	1.63 × 10^−7^	-	>0.05
*MAPK13*	-	>0.05	−1.53	4.65 × 10^−3^
*MAPK14*	0.46	1.19 × 10^−4^	-	>0.05
*NFKBIA*	0.45	3.95 × 10^−6^	0.51	3.75 × 10^−13^
*PTGS2*	-	>0.05	−2.09	1.09 × 10^−6^
*RELA*	-	>0.05	−0.28	1.04 × 10^−2^
*TAB1*	−1.62	1.70 × 10^−10^	-	>0.05
*TAB2*	−0.34	4.29 × 10^−14^	-	>0.05
*TICAM1*	1.34	4.14 × 10^−7^	−0.68	1.00 × 10^−3^
*TIRAP*	-	>0.05	−1.77	2.29 × 10^−4^
*TLR4*	5.32	5.77 × 10^−7^	−0.59	1.02 × 10^−2^
*TOLLIP*	-	>0.05	0.37	2.13 × 10^−2^
*TRAF3*	0.67	2.02 × 10^−9^	−0.29	2.72 × 10^−3^
*TRAF6*	0.52	4.75 × 10^−2^	-	>0.05

The fold change for the analysis of CTR vs. LPS was computed as log_2_ (LPS/CTR). The fold change for the analysis of LPS vs. LPS + LYO was computed as log_2_ (LPS + LYO/LPS). The hyphen symbol in the fold change column is used when *q*-value > 0.05.

**Table 2:** Differentially expressed genes between control cells and human periodontal ligament stem cells (hPDLSCs) treated with lipopolysaccharide (LPS) and between hPDLSCs treated with LPS compared to hPDLSCs treated with LPS and LYO, identified as “Chemokine ligands” and “Interleukins” in the HGNC database.

Gene	CTR vs. LPS Fold Change	CTR vs. LPS *q*-Value	LPS vs. LPS-LYO Fold Change	LPS vs. LPS-LYO *q*-Value
*CCL11*	6.06	6.98 × 10^−5^	−1.65	1.98 × 10^−3^
*CCL2*	5.31	6.28 × 10^−7^	-	>0.05
*CCL26*	4.53	3.88 × 10^−3^	−2.70	3.68 × 10^−2^
*CCL28*	4.53	3.88 × 10^−3^	-	>0.05
*CCL5*	4.59	1.98 × 10^−5^	−1.51	1.97 × 10^−5^
*CXCL1*	2.82	1.17 × 10^−2^	-	>0.05
*CXCL10*	5.60	2.55 × 10^−4^	−1.78	6.58 × 10^−3^
*CXCL16*	4.16	8.55 × 10^−3^	-	>0.05
*CXCL8*	7.21	1.66 × 10^−6^	−2.39	8.73 × 10^−9^
*IL12A*	7.97	1.04 × 10^−7^	−1.98	9.80 × 10^−13^
*IL24*	4.53	3.88 × 10^−3^	−5.15	2.70 × 10^−3^
*IL32*	6.85	5.77 × 10^−6^	−2.03	2.67 × 10^−6^
*IL6*	4.95	3.69 × 10^−11^	−0.61	6.27 × 10^−4^

The fold change for the analysis of CTR vs. LPS was computed as log_2_ (LPS/CTR). The fold change for the analysis of LPS vs. LPS + LYO was computed as log_2_ (LPS + LYO/LPS). The hyphen symbol in the fold change column is used when *q*-value > 0.05.

**Table 3:** Differentially expressed genes between control cells and human periodontal ligament stem cells (hPDLSCs) treated with lipopolysaccharide (LPS) and between hPDLSCs treated with LPS, compared to hPDLSCs treated with LPS and LYO involved in cell migration.

Gene	CTR vs. LPS Fold Change	CTR vs. LPS *q*-Value	LPS vs. LPS-LYO Fold Change	LPS vs. LPS-LYO *q*-Value
*COMP*	−6.51	2.75 × 10^−61^	1.36	1.66 × 10^−2^
*FN1*	−0.82	0	0.20	0
*ITGA2*	−1.87	0	−0.68	9.17 × 10^−24^
*ITGA4*	−1.18	8.92 × 10^−91^	0.33	4.95 × 10^−7^
*ITGB1*	−0.10	8.39 × 10^−14^	0.28	6.94 × 10^−150^
*THBS1*	0.14	6.35 × 10^−89^	0.09	5.50 × 10^−61^
*THBS2*	−1.27	0	−0.41	1.11 × 10^−56^
*THBS3*	−0.82	2.80 × 10^−23^	-	>0.05
*THBS4*	−9.08	3.45 × 10^−18^	-	>0.05
*PDGFA*	-	>0.05	1.30	8.35 × 10^−4^
*PDGFD*	−3.22	1.84 × 10^−41^	1.47	2.66 × 10^−7^
*PDGFRB*	−1.21	0	0.38	3.26 × 10^−28^

The fold change for the analysis of CTR vs. LPS was computed as log_2_ (LPS/CTR). The fold change for the analysis of LPS vs. LPS + LYO was computed as log_2_ (LPS + LYO/LPS). The hyphen symbol in the fold change column is used when *q*-value > 0.05.

## Data Availability

We uploaded the dataset analyzed for this study in the repository Sequence Read Archive (accession number PRJNA791759).
